# Disparities in aortic stenosis and heart failure related mortality trends by sex, race, and geography in United States: A two-decade perspective

**DOI:** 10.1016/j.ahjo.2026.100749

**Published:** 2026-03-12

**Authors:** Mishal Zehra, Syed Ali Hussain, Mujtaba Azhar Siddiqui, Emad Uddin Sajid, Muhammad Murtaza Nadeem, Pashmina Kumari, Tamam Mohamad

**Affiliations:** a Department of Cardiology, Dow University of Health Sciences, 75280, Karachi, Pakistan; b Marian University, Indianapolis, IN, 46222, United States; c Khairpur Medical College, Khairpur, Pakistan; d Henry Ford St. John Hospital, 22101 Moross Rd, Detroit, MI, 48236, United States

**Keywords:** Aortic stenosis, Heart failure, Mortality trends, CDC WONDER, Cardiovascular outcomes, Epidemiology

## Abstract

**Objective:**

This study aimed to evaluate two decades of U.S. mortality patterns in patients with aortic stenosis (AS) and heart failure (HF), focusing on disparities by sex, race, and geography.

**Design:**

Retrospective Study.

**Setting:**

Using the CDC WONDER database, we examined national mortality data from 1999 to 2023.

**Participants:**

Individuals aged ≥45 years with AS and HF were included.

**Main outcome measure:**

Trends in age-adjusted mortality rates (AAMRs) across age, sex, race, geography, and urbanization using Joinpoint regression analysis.

**Results:**

A total of 236,504 deaths were recorded. Overall AAMR increased until 2011 (APC: 0.78, AAMR 9.42), then stabilized through 2023 (APC: 0.07, AAMR 9.45). Mortality rates were consistently higher among males, older adults >65 years, non-Hispanic Whites, rural populations, and the Midwest region. AAMR in older adults was approximately 80 times higher than in middle-aged groups.

**Conclusions:**

Although national mortality rates for AS and HF have stabilized in recent years, they are still on the rise and significant disparities persist across demographic and geographic groups. These findings highlight the need for equitable healthcare access and targeted interventions to reduce preventable cardiovascular deaths.

## Graphical abstract

Central Illustration: Aortic stenosis and heart failure related mortality trends in USA from 1999 to 2023.

**Figure:**
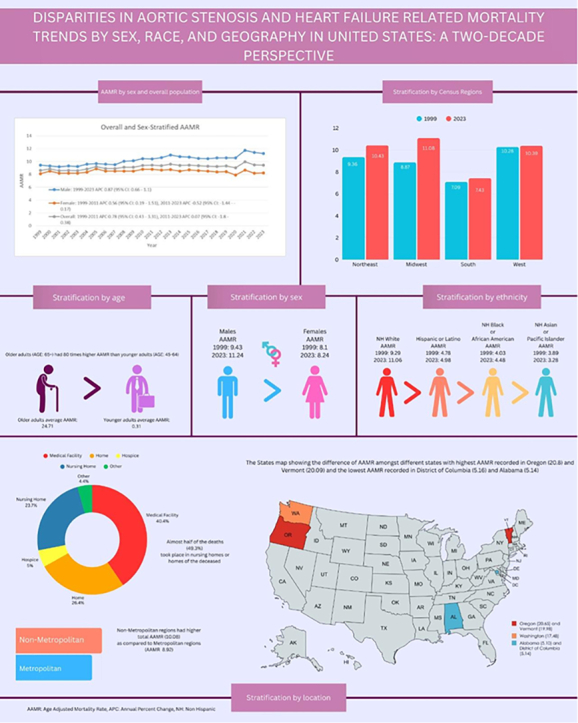

## 1 Introduction

### 1.1 Background

Aortic stenosis (AS) is the most common valvular heart disease in developed countries affecting 25% of adults over 65 years. It primarily results from progressive fibro-calcific remodeling and thickening of the aortic valve leaflets, which gradually leads to severe obstruction of left ventricular (LV) outflow tract [1]. In patients with AS, the increased afterload leads to increased left ventricular mass, resulting in increased LV filling pressures ultimately leading to systolic dysfunction and reduced left ventricular ejection fraction (LVEF). These series of changes leading to maladaptive cardiac remodeling results in a complex clinical syndrome with poor outcomes and high mortality in patients with AS [2].

### 1.2 Current knowledge

Literature reports a steep incline in mortality with reported survival of 2 years after the development of heart failure symptoms [3]. The management of these patients presents significant challenges, as the presence of both conditions can complicate treatment strategies. Furthermore, development of heart failure puts these patients at high - risk for post surgical complications. Despite advancements in medical and surgical therapies including transcatheter aortic valve replacement (TAVR), these patients often experience exacerbated heart failure symptoms despite addressing the valvular pathology [4], [5].

### 1.3 Gaps in knowledge

Although numerous studies have explored the individual mortality patterns of AS and HF across different demographic factors [5], [6], there has been limited attention given to a comprehensive analysis of the evolving mortality trends over extended periods in patients with coexisting AS and HF, particularly considering the relation between various demographic factors. For example, biological differences between sexes and ethnicities, along with variations in access to healthcare, could contribute to differences in mortality rate trends. Similarly, geographic factors and the distinction between metropolitan and non-metropolitan areas may also influence these outcomes.

### 1.4 Aims and rationale

This study seeks to fill this gap in knowledge of the trends in mortality among patients with coexisting AS and HF in the United States over a two-decade period (1999–2023) assessing variations across sex, race/ethnicity, geography, and urbanization levels by using Centers for Disease Control and Prevention, Wide-Ranging Online Data for Epidemiologic Research (CDC WONDER) database. A deeper understanding of these trends could help guide more unbiased evidence-based interventions for high-risk patient populations. Hence, improving survival for these patients.

## 2 Methods

### 2.1 Study setting and population

We collected data for analysis that is available publicly from the CDC WONDER database that included data on the cause of mortality from all deaths in the 50 States and the District of Columbia. We included individuals ≥45 years of age who died naturally due to events directly or associated with AS and HF both. The World Health Organization (WHO) classifies natural death as a disease or injury with events leading directly to death as mentioned on the death certificate by the physician. The data included the age-adjusted mortality rate (AAMR) across different racial groups and gender in the USA from 1999 to 2023 using following International Classification of the Diseases, Tenth Revision (ICD-10) codes to identify Aortic stenosis cases: I35.0 (“Aortic (valve) stenosis”); I06.0 (“Rheumatic aortic stenosis”); and ICD-10 codes I50.0 (“Congestive heart failure”); I50.1 (“Left ventricular failure”); I50.9 (“Heart failure, unspecified”) for heart failure.

### 2.2 Ethics statement

The study was conducted concerning the STROBE (Strengthening the Reporting of Observational Studies in Epidemiology) guidelines. Moreover, this study did not need Institutional review board approval as the anonymized government data, which is publicly available, was used in the study.

### 2.3 Data extraction

We collected mortality data for patients with AS and HF-related deaths from 1999 to 2023. Both AS and HF were selected as multiple causes of death. The data was stratified on the basis of age, sex, race and ethnicity, region, and state. We included patients aged ≥45 years and divided them into 10-year age groups: 45–54 years; 55–64 years; 65–74 years; 75–84 years; 85+ years. The patients were further stratified according to their gender and their respective mortality trends were observed. We also stratified according to the patient race, including White Non-Hispanic (NH) Americans, African NH Americans, Hispanic, Asian/Pacific Islander, and American Indian/Alaskan Native. Furthermore, according to the Census Bureau, we classified regions into Midwest, Northeast, South, and West [6]. We also sub-grouped and analyzed our data into two groups, metropolitan and non-metropolitan areas using the National Center for Health Statistics Urban-Rural Classification Scheme.

### 2.4 Statistical analysis

We analyzed our data on national mortality for patients with AS and HF both by crude death rates and age adjusted mortality rates (AAMRs) per 100,000 population for each year from 1999 to 2023 with the corresponding 95% confidence intervals (CI). The formula used for AAMR is AAMR = Σ (age-specific mortality rate × standard population proportion for each age group). The mortality rates were age-adjusted based on the US standard population from the year 2000. Temporal trends in mortality were assessed to determine changes in slope using Joinpoint Regression Program version 4.7.0.0, which models consecutive linear segments on a log scale, connected by Joinpoints, where the segments converge. We also calculated annual percentage changes (APC) and Average Annual Percentage Changes (AAPCs) with corresponding 95% CIs for the complete time of study, 1999–2023. Slopes were considered increasing or decreasing if the estimated slope differed significantly from zero. The statistical significance was determined by 2-sided t-testing (*P* = 0.05).

## 3 Results

The analysis of mortality trends for patients with AS and HF both revealed significant changes over a period of two decades, from 1999 to 2023 [Central illustration]. The total number of deaths recorded during this period were 236,504. [Table S1].

### 3.1 Overall trends in mortality

**Fig. 1:**
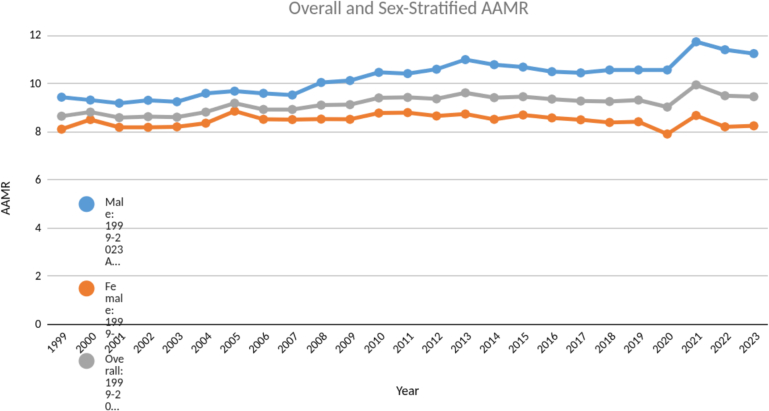
Overall and sex stratified age - adjusted mortality rates (AAMR) from 1999 to 2023.

The AAMR raised steadily from year 1999 till 2011 with an Annual Percent Change (APC) of 0.78 (CI: 0.43–3.31), with highest AAMR being 9.42, where from it increased very slightly all the way till 2023 to a value of 9.45 with an APC of 0.07 (CI: −1.85–0.38) [Fig. 1, Table S1–2] The Average Annual Percent Change (AAPC) of the two decades was found to be 0.42 (CI: 0.2–0.68). Further stratification according to genders, age, race, urbanization status, states and census regions showed stark differences among the groups. To provide broader epidemiologic context, we evaluated population-level AAMRs for HF and AS across the study period. From 1999 to 2023, HF mortality was consistently and substantially higher than AS mortality. The mean AAMR for HF was approximately 260 deaths per 100,000 population, compared with approximately 21 deaths per 100,000 population for AS, representing nearly a twelve-fold difference in mortality burden.

HF mortality demonstrated a wide range of AAMRs over time, with the highest rate observed in 1999 (approximately 299 deaths per 100,000), followed by a gradual decline over subsequent years. In contrast, AS mortality remained relatively stable throughout the study period, fluctuating within a narrow range of approximately 20 to 22 deaths per 100,000 population, with a modest peak near 22 deaths per 100,000. These findings highlight distinct temporal mortality patterns and differing population-level disease burdens between HF and AS. [Table S8].

### 3.2 Gender and age wise stratification

**Fig. 2:**
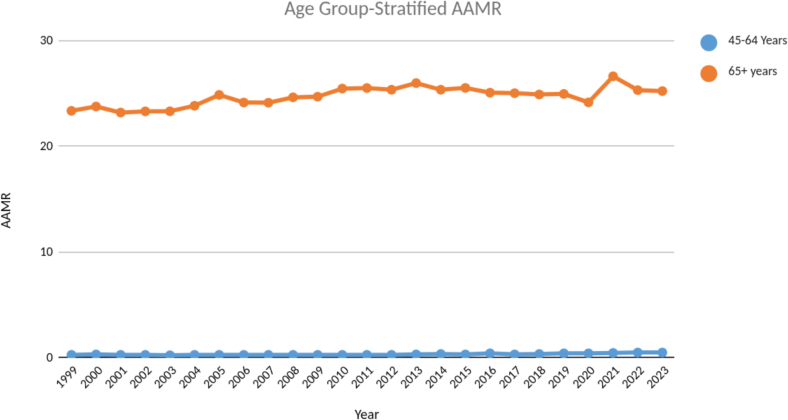
Age - adjusted mortality rates (AAMR) stratified by age - groups (45–64 years and 65 + years) from 1999 to 2023.

The AAMR was consistently higher for males throughout the study period with highest AAMR being 11.73 recorded during the year 2021, whereas the highest AAMR for females was found to be 8.79 during the year 2011. There was a steady incline in AAMR from 1999 till 2011 for females with an APC of 0.56 (CI: 0.19–1.51) which then declined slowly till the year 2023 with an APC of −0.52 (CI: −1.44 - -0.17). For males the AAMR had a steady incline throughout the study period from 1999 to 2023, with an APC of 0.87 (CI: 0.66–1.1) [Fig. 1, Table S1–2]. The age groups were stratified further into two categories; middle-aged (45–64 years) and older adults (65+ years). This stratification revealed about 80 times higher AAMR in the older adults group as compared to the middle-aged group (Average AAMR 24.71 vs. 0.31 for older adults and middle-aged group respectively). (Fig. 2).

### 3.3 Racial stratification

**Fig. 3:**
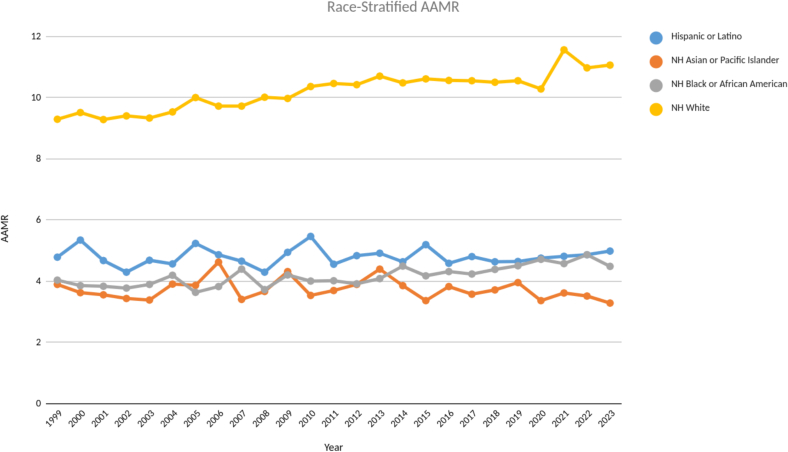
Age - adjusted mortality rates (AAMR) stratified by race from 1999 to 2023.

Among the races, Non-Hispanic (NH) Whites had significantly higher AAMR throughout the two decades with highest AAMR measured at 11.56 for the year 2021 and even the lowest AAMR of 9.28 was significantly higher than the highest AAMRs of 5.46, 4.86 and 4.62 for Hispanic or Latino, NH Black or African Americans and NH Asian or Pacific Islanders, respectively. APC trends were stable for Hispanic or Latino race, but for NH Whites and NH Blacks, it raised steadily all the way to 2023 with NH White having an APC of 0.74 (CI: 0.58–0.9) and NH Black having an APC of 0.88 (CI: 059–1.21) (Fig. 3, Table S1, S3).

### 3.4 State - wise distribution

The highest AAMR was found for the state of Oregon with a value of 20.8 followed shortly by Vermont at 20.09 while the lowest were recorded in the states of District of Columbia and Alabama at 5.16 and 5.14, respectively. [Table S4].

### 3.5 Urbanization

On the basis of urbanization, Non-metropolitan regions had significantly higher AAMRs throughout the whole time period while the highest AAMR for both metropolitan and non-metropolitan regions was found to be during the year 2013 at 9.39 and 10.97, respectively. The trend for non-metropolitan regions increased in two sets, one being a steady incline from 1999 to 2007 (APC 0.75, CI: 1.27–1.59) and the second one being a sharp incline from 2007 to 2013 (APC 1.81, CI: 1.27–3.48), followed by a slow decline from 2013 to 2020 (APC -0.17, CI: −1.24–1.46). (Table S5).

### 3.6 Regional stratification

**Fig. 4:**
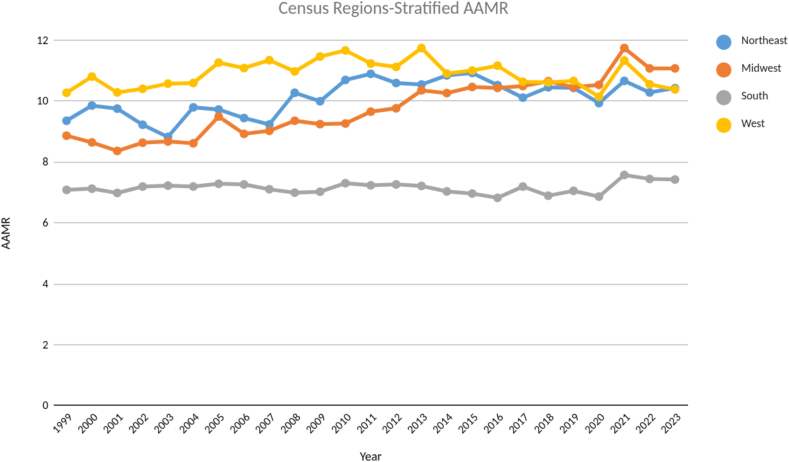
Age - adjusted mortality rates (AAMR) stratified by census regions from 1999 to 2023.

Lastly among the census regions the lowest AAMR throughout the study was found to be in the South whereas till 2013, the West had the highest AAMR with a peak of 11.75 during the same year. From thereon till 2018 the AAMR of West, Midwest and Northeast were similar to each other and the year 2023 culminating with Midwest having the highest AAMR of 11.08 among the census regions correlating with the steady incline in AAMR in the Midwest over 20 years with an APC of 1.25 (CI: 1.06–1.45). (Fig. 4, Table S6).

## 4 Discussion

In this national longitudinal analysis of U.S. mortality data from 1999 to 2023, we observed that deaths attributed to the coexistence of AS and HF have continued to increase modestly over the past two decades. Although the overall AAMR increased only slightly during the more recent years, the long-term AAPC remained positive, indicating persistent disease burden in this high-risk population. The pattern suggests that, despite advances in the diagnosis and treatment of AS and HF, the mortality burden for AS patients with concurrent HF remains substantial. Moreover, the nearly twelve-fold higher overall HF mortality as compared to AS alone reflects the broader prevalence and multifactorial etiology of HF. The comparative population-level trends provide important context for interpreting our findings. While HF mortality has demonstrated a gradual decline over time, AS-related mortality has remained comparatively unchanged, potentially reflecting the progressive nature of valvular degeneration, delayed diagnosis, or disparities in access to definitive valve therapies.

### 4.1 Temporal mortality trends

AS is a common valvular disorder which is associated with higher overall mortality if left untreated. When it is associated with HF, the overall 5 - year survival rate is less than 50% without aortic valve replacement (AVR). Literature review reports that patients with AS and HF combined have higher overall mortality as compared to patients with HF alone [7]. Increased afterload due to LV outflow tract obstruction, leads to cardiac remodeling, altered hemodynamics, thus resulting in adverse outcomes in patients with concomitant HF [2]. Our study findings also align with the previous studies findings and report higher mortality rates with modest increase over the two decades among patients with AS and HF combined. [Fig. 1, Table S3] Although the AAPC remained positive, there had been a relative stabilization in AAMR in recent years. This could be attributed to the advancements in the medical and device therapies for both AS and HF. This includes widespread use of HF therapies such as b-blockers, renin angiotensin aldosterone system inhibitors (RAASi), sodium glucose transport 2 inhibitors (SGLT2i), mineralocorticoid receptor antagonists (MRA) which have shown mortality benefit in HF patients. Furthermore, additional contributors such as earlier detection of AS, increased use of echocardiography, improved peri-procedural care for valve patients, evolving coding and diagnostic practices may have resulted in relative stabilization of mortality trends. Moreover, an increasing number transcatheter aortic valve replacement (TAVR) procedures are now being performed in patients in recent years, who were previously deemed at high - risk of surgery due to concomitant HF. This may also have contributed to the relative stabilization over the recent years. Studies have reported a modest improvement in LVEF following TAVR [8], [9].

### 4.2 Gender disparities

Consistent with the existing literature, significant gender disparity with notably higher mortality rates in males as compared to females was observed in our study [Fig. 2]. Multiple factors may contribute to this. Anatomically, women tend to have smaller left ventricular outflow tracts and valve annuli, which influence the hemodynamic severity of AS and the decision-making process for interventions such as TAVR or surgical AVR (SAVR) [10]. Despite this, women have been shown to have better clinical outcomes after TAVR than men, potentially due to better vascular compliance and lower burden of aortic valve calcification [11], [12], [13]. In the context of HF, sex contributes to phenotypic heterogeneity with women exhibiting higher rates of concentric LV remodeling as compared to men showing eccentric remodeling and LV dilatation which contributes to adverse cardiovascular outcomes [14].

### 4.3 Age - related trends

Advanced age is a well known risk factor for cardiovascular disease. Our study also reports an increased mortality rate among elderly, particularly those aged ≥85 years. This age group is inherently more vulnerable due to multiple comorbidities, frailty, and reduced physiological reserve [15]. Furthermore, AS and HF both progress insidiously in older adults and may remain underdiagnosed or untreated until the disease reaches an advanced stage, contributing to poor outcomes and higher rates of procedural complications during SAVR or TAVI [16].

### 4.4 Racial disparities

Our analysis revealed significant racial disparity with NH whites accounting for the highest mortality among all racial groups [Fig. 3, Table S4]. Although previous studies have reported that racial minorities experience higher mortality rates due to disparities in socioeconomic status, healthcare access, quality of care, and comorbidity burden, but the fact that NH whites showed the highest mortality rates in our study suggest complex underlying factors. This could be attributed to the demographic structure as NH White populations in the U.S. are older on average, and both calcific AS and HF–related mortality increase steeply with age as indicated in our age wise stratification results. Thus, these mortality rates may disproportionately reflect population age distribution rather than intrinsic risk differences. Additionally, we note that calcific AS has a higher baseline prevalence among NH White individuals, which may also contribute to higher AS and HF mortality independent of access or socioeconomic factors [17], [18]. Moreover, potential coding and reporting differences may also account for the differences in mortality trends. Variability in death certificate completion, diagnostic labeling, and recognition of AS—known limitations of administrative datasets—may generate apparent racial differences that do not reflect true disease burden. Therefore, these findings highlight the need for further focused research to account for these disparities.

### 4.5 Geographic and regional disparities

A notable regional gap was also noted, with the Midwest region exhibiting the highest AAMRs over the past two decades [Table S6]. Additionally, states like Oregon and Vermont consistently reported among the highest state-specific AAMRs [Fig. 4, Table S5]. These disparities could be attributed to variable coding methods across states, particularly in predominantly rural populations, differences in time to diagnosis and recognition of AS or HF, aging population, which may disproportionately influence the mortality rates. General healthcare access differences may also contribute to these disparities such as time to specialty evaluation or structural heart intervention referral patterns [19]. Differences in local healthcare policy, funding, and public health initiatives may also contribute to these trends. These observations emphasize the need for more focused research and data on TAVR centers availability, structural heart program distribution, cardiologist density and socioeconomic indicators to address these disparities.

### 4.6 Urban rural divide

Our findings indicate that non-metropolitan areas had higher AAMRs compared to metropolitan areas [Table S7]. This urban–rural divide in cardiovascular outcomes has been consistently documented and is largely attributed to limited access to specialized cardiac care, non - availability of TAVR capable centers in the vicinity, longer travel distances to tertiary care centers, and socioeconomic disparities that disproportionately affect rural populations. Moreover, awareness of AS and HF symptoms may be lower in rural communities, contributing to delays in diagnosis and timely referral for life-saving procedures like TAVR [20]. Therefore, public health initiatives, awareness campaigns and timely referral of patients to specialized centers is paramount to reduce the mortality burden.

### 4.7 Future prospects

Our study highlights the combined effect of AS and HF on the mortality trends. Although the temporal trends show a steady state decline, there is still a higher reported mortality with significant age, gender, geographic and regional disparities demanding for future prospective studies to identify the potential confounding factors and guide management strategies. This study also emphasizes the need for strict implementation of guideline directed medical and surgical intervention in underserved areas and provision of equitable healthcare access in rural areas to reduce the mortality burden. Furthermore, many large scale clinical trials and meta – analysis have demonstrated mortality benefit with the early use of SGLT-2 inhibitors, a cardioprotective drug, in patients at risk of HF and in post - TAVI patients [21], [22]. However, the role of SGLT-2 inhibitors in patients with AS alone is still not widely studied. Studies have shown that in patients with AS, SGLT-2 is expressed in the myocardium and associated with fibrosis, inflammation and energetic dysfunction [23]. Therefore, further research in this area is required for proactive management and early introduction of this cardioprotective drug to potentially improve outcomes, reduce mortality rate and lower healthcare costs in this population.

Although this study was specifically designed to characterize national mortality trends among individuals with both AS and HF, it is important to note that this comparison reflects overall disease-specific mortality trends rather than a direct risk comparison between HF patients with and without AS. Future investigations incorporating individual-level comparative analyses with adjustment for comorbidities, demographic factors, and treatment variables are warranted to better define the incremental mortality risk attributable to AS among patients with HF. Moreover, these trends should be validated using clinical registry datasets in future research.

## 5 Limitations

Despite the large sample size and national representation, this study has several important limitations. First, it relies on population-level mortality data derived from death certificates, which may be subject to misclassification and can lead to under- or over-reporting of aortic stenosis and heart failure. Temporal trends may be influenced by greater recognition of AS and improved coding practices due to increased clinical attention to the disease. Geographic variation may reflect differences in state-level coding culture, autopsy practices, or cause-of-death attribution, not solely differences in true mortality. Second, CDC WONDER provides only aggregated data, which limits the ability to draw individual-level inferences and introduces the potential for ecological fallacy; therefore, observed associations at the population level cannot be assumed to apply to individual patients. Additionally, only univariate analysis can be performed given the present dataset. The dataset lacks key clinical details such as aortic stenosis severity, type of heart failure (preserved vs. reduced ejection fraction), treatment strategies, and procedural history. Information on comorbidities—including coronary artery disease, arrhythmias, metabolic disorders, or chronic inflammatory conditions—is also unavailable, restricting the ability to adjust for potential confounders. As a result, while our findings reflect national mortality trends among patients documented to have both AS and HF, they should not be interpreted as causal or patient-level risk assessments. Future studies using electronic health records and detailed clinical registries are needed to validate these findings and explore underlying mechanisms contributing to mortality in this population.

## 6 Conclusion

This study highlights the evolving mortality trends in patients with AS and HF. The observed stabilization in mortality rates over the recent years is although encouraging, persistent disparities with higher mortality rates in males, elderly, NH whites, Midwest region and rural areas highlight the urgent need for equitable, accessible, and targeted healthcare strategies to reduce preventable deaths in this high-risk population.

## CRediT authorship contribution statement

**Mishal Zehra:** Writing – review & editing, Writing – original draft, Conceptualization. **Syed Ali Hussain:** Writing – original draft, Formal analysis, Data curation. **Mujtaba Azhar Siddiqui:** Writing – original draft, Investigation. **Emad Uddin Sajid:** Writing – original draft, Methodology. **Muhammad Murtaza Nadeem:** Writing – original draft. **Pashmina Kumari:** Writing – original draft. **Tamam Mohamad:** Validation, Supervision.

## Ethics statement

This ethics statement affirms that the research adhered to the highest ethical standards and guidelines established by regulatory bodies. The study did not require consent from participants or IRB approval as the data was abstracted from an anonymized publicly available database, conflicts of interest were denied, high morals were upheld, data integrity and transparency were ensured, authorship criteria were outlined, and contributions were acknowledged appropriately.

## Funding

None.

## Declaration of competing interest

None.

## Data Availability

The data that support the findings of this study are publicly available through the CDC WONDER database. Any additional data related to this study will be made available in accordance with journal policies.

## Appendix A Supplementary data

Supplementary tables Image 1

## References

[1] Lindman B.R., Clavel M.A., Mathieu P., Iung B., Lancellotti P., Otto C.M., Pibarot P. (2016 Mar 3). Calcific aortic stenosis. Nat. Rev. Dis. Primers.

[2] Mengi S., Januzzi J.L., Cavalcante J.L., Avvedimento M., Galhardo A., Bernier M., Rodés-Cabau J. (2024). Aortic stenosis, heart failure, and aortic valve replacement. JAMA Cardiol..

[3] Frank S., Johnson A., Ross J. (1973 Jan). Natural history of valvular aortic stenosis. Br. Heart J..

[4] Baumgartner H., Falk V., Bax J.J., De Bonis M., Hamm C., Holm P.J., Iung B., Lancellotti P., Lansac E., Munoz D.R., Rosenhek R. (2018). 2017 ESC/EACTS Guidelines for the management of valvular heart disease. Polish Heart J. (Kardiologia Polska).

[5] Nishimura R.A., Otto C.M., Bonow R.O., Carabello B.A., Erwin J.P., Fleisher L.A., Jneid H., Mack M.J., McLeod C.J., O’Gara P.T., Rigolin V.H. (2017 Jul 11). 2017 AHA/ACC focused update of the 2014 AHA/ACC guideline for the management of patients with valvular heart disease: a report of the American College of Cardiology/American Heart Association Task Force on Clinical Practice Guidelines. J. Am. Coll. Cardiol..

[6] Ingram D.D., Franco S.J. (2014).

[7] Jean G., Van Mieghem N.M., Gegenava T., van Gils L., Bernard J., Geleijnse M.L., Vollema E.M., El Azzouzi I., Spitzer E., Delgado V., Bax J.J. (2021 Jun 8). Moderate aortic stenosis in patients with heart failure and reduced ejection fraction. J. Am. Coll. Cardiol..

[8] Parikh P.B., Mack M., Stone G.W., Anker S.D., Gilchrist I.C., Kalogeropoulos A.P., Packer M., Skopicki H.A., Butler J. (2024 Feb). Transcatheter aortic valve replacement in heart failure. Eur. J. Heart Fail..

[9] Sapna F.N., Raveena F.N., Chandio M., Bai K., Sayyar M., Varrassi G., Khatri M., Kumar S., Mohamad T. (2023 Oct 4). Advancements in heart failure management: a comprehensive narrative review of emerging therapies. Cureus.

[10] Hamdan A., Barbash I., Schwammenthal E., Segev A., Kornowski R., Assali A., Shaviv E., Fefer P., Goitein O., Konen E., Guetta V. (2017 Mar 1). Sex differences in aortic root and vascular anatomy in patients undergoing transcatheter aortic valve implantation: a computed-tomographic study. J. Cardiovasc. Comput. Tomogr..

[11] Goel H., Kumar A., Garg N., Mills J.D. (2021 Jan 1). Men are from mars, women are from venus: factors responsible for gender differences in outcomes after surgical and trans-catheter aortic valve replacement. Trends Cardiovasc. Med..

[12] Thaden J.J., Nkomo V.T., Suri R.M., Maleszewski J.J., Soderberg D.J., Clavel M.A., Pislaru S.V., Malouf J.F., Foley T.A., Oh J.K., Miller J.D. (2016 Feb 21). Sex-related differences in calcific aortic stenosis: correlating clinical and echocardiographic characteristics and computed tomography aortic valve calcium score to excised aortic valve weight. Eur. Heart J..

[13] Aggarwal S.R., Clavel M.A., Messika-Zeitoun D., Cueff C., Malouf J., Araoz P.A., Mankad R., Michelena H., Vahanian A., Enriquez-Sarano M. (2013 Jan). Sex differences in aortic valve calcification measured by multidetector computed tomography in aortic stenosis. Circ. Cardiovasc. Imaging.

[14] Shi K., Zhang G., Fu H., Li X.M., Jiang L., Gao Y., Qian W.L., Shen L.T., Xu H.Y., Li Y., Guo Y.K. (2024 Jul 22). Sex differences in clinical profile, left ventricular remodeling and cardiovascular outcomes among diabetic patients with heart failure and reduced ejection fraction: a cardiac-MRI-based study. Cardiovasc. Diabetol..

[15] Ramos M., Quezada M., Ayala R., Gómez-Pavón F.J., Jaramillo J., Calderón-Domínguez M., Toro R. (2021 Feb 1). Asymptomatic aortic stenosis in a geriatric population. The role of frailty and comorbidity in mortality. Rev. Española Cardiol. (English Ed.).

[16] Díez-Villanueva P., Jiménez-Méndez C., Alfonso F. (2021 Mar 28). Heart failure in the elderly. J. Geriatric Cardiol. JGC.

[17] Enard K.R., Coleman A.M., Yakubu R.A., Butcher B.C., Tao D., Hauptman P.J. (2023 Feb 7). Influence of social determinants of health on heart failure outcomes: a systematic review. J. Am. Heart Assoc..

[18] Wilson J.B., Jackson L.R., Ugowe F.E., Jones T., Yankey G.S., Marts C., Thomas K.L. (2020 Jan 27). Racial and ethnic differences in treatment and outcomes of severe aortic stenosis: a review. Cardiovasc. Interv. Ther..

[19] Parcha V., Kalra R., Suri S.S., Malla G., Wang T.J., Arora G., Arora P. (2021 Jul 1). Mayo Clinic Proceedings.

[20] Pierce J.B., Shah N.S., Petito L.C., Pool L., Lloyd-Jones D.M., Feinglass J., Khan S.S. (2021 Mar 3). Trends in heart failure-related cardiovascular mortality in rural versus urban United States counties, 2011–2018: a cross-sectional study. PLoS One.

[21] Raposeiras-Roubin S., Amat-Santos I.J., Rossello X., González Ferreiro R., González Bermúdez I., Lopez Otero D., Nombela-Franco L., Gheorghe L., Diez J.L., Baladrón Zorita C., Baz J.A. (2025 Apr 10). Dapagliflozin in patients undergoing transcatheter aortic-valve implantation. N. Engl. J. Med..

[22] Packer M., Anker S.D., Butler J., Filippatos G., Ferreira J.P., Pocock S.J., Carson P., Anand I., Doehner W., Haass M., Komajda M. (2021 Jan 26). Effect of empagliflozin on the clinical stability of patients with heart failure and a reduced ejection fraction: the EMPEROR-reduced trial. Circulation.

[23] Karakasis P., Theofilis P., Patoulias D., Vlachakis P.K., Pamporis K., Sagris M., Ktenopoulos N., Kassimis G., Antoniadis A.P., Fragakis N. (2025 May 8). Sodium–glucose cotransporter 2 inhibitors in aortic stenosis: toward a comprehensive cardiometabolic approach. Int. J. Mol. Sci..

