# Supplementary material for: Disparities in aortic stenosis and heart failure related mortality trends by sex, race, and geography in United States: A two-decade perspective
Source: Am Heart J Plus. 2026 Mar 12;64:100749. doi: 10.1016/j.ahjo.2026.100749 (PMC12999315; doi:10.1016/j.ahjo.2026.100749)
Supplement: Supplementary file 1 — Supplementary tables [file mmc1.docx]

**Supplemental Table 1:** Aortic Stenosis and Heart Failure–related Deaths, Stratified by Sex and Race, in Older Adults in the United States, 1999 to 2023

|  | Deaths | | | | | | | |
| --- | --- | --- | --- | --- | --- | --- | --- | --- |
| Year | **Overall** | **Women** | **Men** | **NH White** | **NH Black or African American** | **NH Asian or Pacific Islander** | **Hispanic or Latino** | **Population** |
| 1999 | 8138 | 5105 | 3033 | 7575 | 291 | 63 | 177 | 95153686 |
| 2000 | 8435 | 5412 | 3023 | 7854 | 283 | 65 | 206 | 96944389 |
| 2001 | 8360 | 5291 | 3069 | 7780 | 284 | 66 | 196 | 99781854 |
| 2002 | 8504 | 5352 | 3152 | 7947 | 276 | 71 | 183 | 102217733 |
| 2003 | 8627 | 5423 | 3204 | 8018 | 289 | 75 | 211 | 104692428 |
| 2004 | 8953 | 5578 | 3375 | 8302 | 312 | 91 | 220 | 107138553 |
| 2005 | 9571 | 6033 | 3538 | 8895 | 280 | 97 | 263 | 109787199 |
| 2006 | 9554 | 5936 | 3618 | 8830 | 300 | 125 | 257 | 112380379 |
| 2007 | 9775 | 6063 | 3712 | 9031 | 357 | 94 | 266 | 114894084 |
| 2008 | 10203 | 6177 | 4026 | 9487 | 311 | 112 | 254 | 117395131 |
| 2009 | 10463 | 6295 | 4168 | 9611 | 365 | 144 | 312 | 119895863 |
| 2010 | 11010 | 6589 | 4421 | 10131 | 358 | 123 | 364 | 121757429 |
| 2011 | 11413 | 6820 | 4593 | 10542 | 368 | 140 | 329 | 124174484 |
| 2012 | 11625 | 6813 | 4812 | 10675 | 371 | 157 | 372 | 126000296 |
| 2013 | 12207 | 7016 | 5191 | 11146 | 402 | 196 | 411 | 127788037 |
| 2014 | 12194 | 6936 | 5258 | 11075 | 465 | 186 | 411 | 129779643 |
| 2015 | 12568 | 7222 | 5346 | 11381 | 442 | 177 | 497 | 131826832 |
| 2016 | 12641 | 7252 | 5389 | 11429 | 474 | 213 | 466 | 133494018 |
| 2017 | 12827 | 7301 | 5526 | 11557 | 484 | 213 | 510 | 135229289 |
| 2018 | 13048 | 7281 | 5767 | 11701 | 518 | 236 | 523 | 136335528 |
| 2019 | 13310 | 7375 | 5935 | 11892 | 551 | 267 | 546 | 137381702 |
| 2020 | 13078 | 7002 | 6076 | 11602 | 596 | 237 | 585 | 138429175 |
| 2021 | 13506 | 7112 | 6394 | 12012 | 551 | 248 | 585 | 139339453 |
| 2022 | 13854 | 7325 | 6529 | 12224 | 614 | 265 | 624 | 140311934 |
| 2023 | 13609 | 7081 | 6528 | 12012 | 574 | 253 | 647 | 141596553 |
| Total | 277473 | 161790 | 115683 | 252709 | 10116 | 3914 | 9415 |  |

NH, non-Hispanic.

**Supplemental Table 2:** Overall and Sex‐Stratified Aortic Stenosis and Heart Failure–related Age-Adjusted Mortality Rates per 10,000 in Older Adults in the United States, 1999 to 2023

|  | Age-Adjusted Rate (95% CI) | | |
| --- | --- | --- | --- |
| Year | **Men** | **Women** | **Overall** |
| 1999 | 9.43 (9.09 - 9.78) | 8.10 (7.88 - 8.33) | 8.64 (8.45 - 8.82) |
| 2000 | 9.31 (8.98 - 9.65) | 8.50 (8.28 - 8.73) | 8.81 (8.62 - 9.00) |
| 2001 | 9.18 (8.85 - 9.51) | 8.18 (7.96 - 8.40) | 8.58 (8.39 - 8.76) |
| 2002 | 9.30 (8.97 - 9.63) | 8.18 (7.96 - 8.40) | 8.62 (8.43 - 8.80) |
| 2003 | 9.24 (8.91 - 9.56) | 8.20 (7.98 - 8.42) | 8.60 (8.42 - 8.78) |
| 2004 | 9.59 (9.27 - 9.92) | 8.35 (8.13 - 8.57) | 8.81 (8.63 - 8.99) |
| 2005 | 9.68 (9.36 - 10.00) | 8.85 (8.63 - 9.08) | 9.18 (9.00 - 9.37) |
| 2006 | 9.59 (9.27 - 9.90) | 8.51 (8.29 - 8.72) | 8.92 (8.74 - 9.10) |
| 2007 | 9.52 (9.22 - 9.83) | 8.50 (8.28 - 8.71) | 8.92 (8.74 - 9.09) |
| 2008 | 10.04 (9.73 - 10.35) | 8.52 (8.30 - 8.73) | 9.10 (8.92 - 9.28) |
| 2009 | 10.12 (9.81 - 10.43) | 8.51 (8.30 - 8.72) | 9.12 (8.95 - 9.30) |
| 2010 | 10.46 (10.15 - 10.77) | 8.77 (8.55 - 8.98) | 9.40 (9.23 - 9.58) |
| 2011 | 10.41 (10.10 - 10.71) | 8.79 (8.58 - 9.00) | 9.42 (9.25 - 9.59) |
| 2012 | 10.59 (10.29 - 10.89) | 8.65 (8.44 - 8.86) | 9.36 (9.19 - 9.53) |
| 2013 | 10.99 (10.69 - 11.29) | 8.73 (8.52 - 8.94) | 9.61 (9.44 - 9.79) |
| 2014 | 10.78 (10.49 - 11.08) | 8.51 (8.30 - 8.71) | 9.41 (9.25 - 9.58) |
| 2015 | 10.68 (10.39 - 10.97) | 8.69 (8.48 - 8.89) | 9.45 (9.28 - 9.62) |
| 2016 | 10.49 (10.21 - 10.77) | 8.57 (8.37 - 8.77) | 9.35 (9.18 - 9.51) |
| 2017 | 10.44 (10.16 - 10.72) | 8.49 (8.29 - 8.69) | 9.27 (9.11 - 9.43) |
| 2018 | 10.56 (10.29 - 10.84) | 8.38 (8.18 - 8.57) | 9.25 (9.09 - 9.41) |
| 2019 | 10.56 (10.29 - 10.83) | 8.41 (8.21 - 8.60) | 9.31 (9.15 - 9.47) |
| 2020 | 10.56 (10.29 - 10.83) | 7.90 (7.71 - 8.09) | 9.02 (8.86 - 9.17) |
| 2021 | 11.73 (11.44 - 12.02) | 8.67 (8.47 - 8.88) | 9.94 (9.77 - 10.11) |
| 2022 | 11.40 (11.12 - 11.68) | 8.20 (8.01 - 8.39) | 9.49 (9.33 - 9.64) |
| 2023 | 11.24 (10.96 - 11.51) | 8.24 (8.05 - 8.44) | 9.45 (9.29 - 9.61) |

**Supplemental Table 3:** Aortic Stenosis and Heart Failure–related Age-Adjusted Mortality Rates per 10,000, Stratified by Race in Older Adults in the United States, 1999 to 2023

|  | Age-Adjusted Rate (95% CI) | | | |
| --- | --- | --- | --- | --- |
| Year | **NH White** | **NH Black or African American** | **Hispanic or Latino** | **NH Asian or Pacific Islander** |
| 1999 | 9.29 (9.08 - 9.50) | 4.03 (3.57 - 4.50) | 4.78 (4.06 - 5.49) | 3.89 (2.97 - 5.01) |
| 2000 | 9.51 (9.30 - 9.72) | 3.85 (3.40 - 4.31) | 5.34 (4.60 - 6.08) | 3.62 (2.78 - 4.65) |
| 2001 | 9.28 (9.08 - 9.49) | 3.83 (3.38 - 4.28) | 4.67 (4.00 - 5.34) | 3.55 (2.74 - 4.54) |
| 2002 | 9.40 (9.19 - 9.61) | 3.77 (3.32 - 4.21) | 4.29 (3.66 - 4.93) | 3.43 (2.67 - 4.34) |
| 2003 | 9.33 (9.12 - 9.53) | 3.89 (3.44 - 4.34) | 4.68 (4.04 - 5.33) | 3.38 (2.65 - 4.25) |
| 2004 | 9.53 (9.32 - 9.73) | 4.19 (3.73 - 4.66) | 4.56 (3.95 - 5.18) | 3.90 (3.13 - 4.80) |
| 2005 | 10.00 (9.79 - 10.21) | 3.63 (3.20 - 4.06) | 5.23 (4.59 - 5.88) | 3.86 (3.12 - 4.72) |
| 2006 | 9.72 (9.51 - 9.92) | 3.82 (3.39 - 4.25) | 4.86 (4.25 - 5.46) | 4.62 (3.80 - 5.43) |
| 2007 | 9.72 (9.52 - 9.93) | 4.39 (3.93 - 4.85) | 4.65 (4.08 - 5.21) | 3.40 (2.75 - 4.17) |
| 2008 | 10.01 (9.81 - 10.21) | 3.72 (3.30 - 4.14) | 4.29 (3.75 - 4.82) | 3.66 (2.98 - 4.35) |
| 2009 | 9.97 (9.77 - 10.17) | 4.20 (3.77 - 4.64) | 4.94 (4.39 - 5.50) | 4.31 (3.59 - 5.02) |
| 2010 | 10.36 (10.16 - 10.57) | 4.00 (3.59 - 4.42) | 5.46 (4.89 - 6.03) | 3.53 (2.90 - 4.16) |
| 2011 | 10.46 (10.26 - 10.66) | 4.01 (3.60 - 4.42) | 4.55 (4.06 - 5.05) | 3.69 (3.08 - 4.31) |
| 2012 | 10.42 (10.22 - 10.62) | 3.91 (3.50 - 4.31) | 4.83 (4.33 - 5.32) | 3.89 (3.28 - 4.50) |
| 2013 | 10.70 (10.50 - 10.90) | 4.08 (3.68 - 4.48) | 4.91 (4.43 - 5.39) | 4.39 (3.78 - 5.01) |
| 2014 | 10.48 (10.28 - 10.68) | 4.49 (4.07 - 4.90) | 4.63 (4.18 - 5.08) | 3.85 (3.30 - 4.41) |
| 2015 | 10.61 (10.42 - 10.81) | 4.17 (3.78 - 4.56) | 5.19 (4.73 - 5.65) | 3.36 (2.86 - 3.86) |
| 2016 | 10.56 (10.37 - 10.76) | 4.31 (3.91 - 4.70) | 4.58 (4.16 - 5.00) | 3.82 (3.30 - 4.34) |
| 2017 | 10.55 (10.36 - 10.75) | 4.23 (3.84 - 4.61) | 4.80 (4.38 - 5.22) | 3.57 (3.08 - 4.05) |
| 2018 | 10.50 (10.31 - 10.69) | 4.38 (4.00 - 4.76) | 4.63 (4.23 - 5.03) | 3.71 (3.23 - 4.18) |
| 2019 | 10.55 (10.36 - 10.74) | 4.50 (4.12 - 4.88) | 4.64 (4.25 - 5.04) | 3.95 (3.47 - 4.43) |
| 2020 | 10.28 (10.09 - 10.47) | 4.71 (4.33 - 5.10) | 4.75 (4.36 - 5.14) | 3.36 (2.93 - 3.79) |
| 2021 | 11.56 (11.35 - 11.77) | 4.57 (4.18 - 4.96) | 4.81 (4.41 - 5.21) | 3.61 (3.16 - 4.06) |
| 2022 | 10.97 (10.78 - 11.16) | 4.86 (4.47 - 5.25) | 4.86 (4.47 - 5.24) | 3.51 (3.08 - 3.93) |
| 2023 | 11.06 (10.86 - 11.26) | 4.48 (4.11 - 4.86) | 4.98 (4.59 - 5.37) | 3.28 (2.87 - 3.68) |

NH = non-Hispanic.

**Supplemental Table 4:** Aortic Stenosis and Heart Failure–related Age-Adjusted Mortality Rates per 10,000, Stratified by States in Older Adults in the United States, 1999 to 2020

| State | Age-Adjusted Rate (95% CI) |
| --- | --- |
| Alabama | 5.10 (4.87 - 5.33) |
| Alaska | 12.59 (11.21 - 13.97) |
| Arizona | 6.52 (6.30 - 6.74) |
| Arkansas | 6.05 (5.75 - 6.35) |
| California | 10.39 (10.27 - 10.51) |
| Colorado | 8.19 (7.88 - 8.49) |
| Connecticut | 9.23 (8.91 - 9.54) |
| Delaware | 9.79 (9.08 - 10.49) |
| District of Columbia | 5.14 (4.49 - 5.79) |
| Florida | 6.17 (6.06 - 6.27) |
| Georgia | 5.56 (5.37 - 5.75) |
| Hawaii | 8.13 (7.65 - 8.61) |
| Idaho | 13.39 (12.73 - 14.05) |
| Illinois | 7.49 (7.32 - 7.65) |
| Indiana | 9.64 (9.37 - 9.90) |
| Iowa | 12.23 (11.85 - 12.61) |
| Kansas | 9.36 (8.99 - 9.73) |
| Kentucky | 6.72 (6.44 - 6.99) |
| Louisiana | 5.86 (5.61 - 6.12) |
| Maine | 13.85 (13.22 - 14.48) |
| Maryland | 8.37 (8.10 - 8.64) |
| Massachusetts | 11.05 (10.79 - 11.30) |
| Michigan | 8.61 (8.41 - 8.80) |
| Minnesota | 12.48 (12.16 - 12.80) |
| Mississippi | 5.84 (5.53 - 6.16) |
| Missouri | 8.59 (8.34 - 8.84) |
| Montana | 10.91 (10.23 - 11.58) |
| Nebraska | 11.19 (10.70 - 11.69) |
| Nevada | 6.70 (6.31 - 7.10) |
| New Hampshire | 14.74 (14.03 - 15.44) |
| New Jersey | 9.86 (9.64 - 10.08) |
| New Mexico | 5.63 (5.26 - 6.00) |
| New York | 7.38 (7.25 - 7.50) |
| North Carolina | 8.96 (8.74 - 9.17) |
| North Dakota | 9.79 (9.07 - 10.52) |
| Ohio | 9.68 (9.50 - 9.87) |
| Oklahoma | 7.03 (6.74 - 7.33) |
| Oregon | 20.65 (20.17 - 21.13) |
| Pennsylvania | 12.47 (12.28 - 12.66) |
| Rhode Island | 10.67 (10.07 - 11.28) |
| South Carolina | 9.03 (8.71 - 9.34) |
| South Dakota | 10.12 (9.44 - 10.81) |
| Tennessee | 8.52 (8.26 - 8.78) |
| Texas | 7.41 (7.28 - 7.55) |
| Utah | 7.66 (7.21 - 8.10) |
| Vermont | 19.98 (18.84 - 21.13) |
| Virginia | 8.18 (7.95 - 8.41) |
| Washington | 17.48 (17.12 - 17.83) |
| West Virginia | 8.08 (7.66 - 8.49) |
| Wisconsin | 11.90 (11.61 - 12.20) |
| Wyoming | 9.60 (8.66 - 10.54) |

**Supplemental Table 5:** Aortic Stenosis and Heart Failure–related Age-Adjusted Mortality Rates per 10,000, Stratified by Urban-Rural Classification in Older Adults in the United States, 1999 to 2020

|  | Age-Adjusted Rate (95% CI) | |
| --- | --- | --- |
| Year | **Metropolitan** | **Nonmetropolitan** |
| 1999 | 8.55 (8.34 - 8.76) | 8.92 (8.50 - 9.35) |
| 2000 | 8.70 (8.49 - 8.91) | 9.31 (8.87 - 9.74) |
| 2001 | 8.44 (8.24 - 8.65) | 9.21 (8.78 - 9.64) |
| 2002 | 8.41 (8.21 - 8.61) | 9.52 (9.08 - 9.96) |
| 2003 | 8.45 (8.25 - 8.65) | 9.17 (8.74 - 9.60) |
| 2004 | 8.71 (8.51 - 8.91) | 9.27 (8.84 - 9.70) |
| 2005 | 9.08 (8.88 - 9.28) | 9.59 (9.15 - 10.02) |
| 2006 | 8.81 (8.61 - 9.01) | 9.55 (9.12 - 9.98) |
| 2007 | 8.72 (8.53 - 8.92) | 9.74 (9.30 - 10.17) |
| 2008 | 8.94 (8.74 - 9.13) | 9.80 (9.37 - 10.23) |
| 2009 | 8.90 (8.71 - 9.09) | 10.06 (9.63 - 10.49) |
| 2010 | 9.30 (9.10 - 9.49) | 9.97 (9.54 - 10.40) |
| 2011 | 9.20 (9.01 - 9.39) | 10.42 (9.99 - 10.86) |
| 2012 | 9.12 (8.93 - 9.31) | 10.69 (10.25 - 11.12) |
| 2013 | 9.35 (9.16 - 9.54) | 10.89 (10.45 - 11.32) |
| 2014 | 9.13 (8.94 - 9.31) | 10.64 (10.21 - 11.07) |
| 2015 | 9.18 (9.00 - 9.36) | 10.85 (10.42 - 11.29) |
| 2016 | 9.09 (8.91 - 9.27) | 10.65 (10.22 - 11.07) |
| 2017 | 9.08 (8.90 - 9.25) | 10.37 (9.95 - 10.78) |
| 2018 | 8.99 (8.82 - 9.17) | 10.54 (10.13 - 10.96) |
| 2019 | 8.97 (8.80 - 9.14) | 10.85 (10.43 - 11.27) |
| 2020 | 8.64 (8.48 - 8.81) | 10.70 (10.29 - 11.12) |
| Total | **8.92 (8.88 - 8.96)** | **10.08 (9.99 - 10.17)** |

**Supplemental Table 6:** Aortic Stenosis and Heart Failure–related Age-Adjusted Mortality Rates per 10,000, Stratified by Census Region in Older Adults in the United States, 1999 to 2023

| Census Region | Year | Age-Adjusted Rate (95% CI) |
| --- | --- | --- |
| Northeast | 1999 | 9.36 (8.94 - 9.77) |
| Northeast | 2000 | 9.86 (9.44 - 10.29) |
| Northeast | 2001 | 9.76 (9.34 - 10.18) |
| Northeast | 2002 | 9.23 (8.82 - 9.63) |
| Northeast | 2003 | 8.83 (8.43 - 9.22) |
| Northeast | 2004 | 9.80 (9.38 - 10.21) |
| Northeast | 2005 | 9.73 (9.33 - 10.14) |
| Northeast | 2006 | 9.45 (9.05 - 9.85) |
| Northeast | 2007 | 9.24 (8.85 - 9.63) |
| Northeast | 2008 | 10.28 (9.87 - 10.69) |
| Northeast | 2009 | 10.00 (9.60 - 10.40) |
| Northeast | 2010 | 10.70 (10.29 - 11.11) |
| Northeast | 2011 | 10.90 (10.49 - 11.31) |
| Northeast | 2012 | 10.60 (10.20 - 11.00) |
| Northeast | 2013 | 10.55 (10.16 - 10.95) |
| Northeast | 2014 | 10.85 (10.45 - 11.25) |
| Northeast | 2015 | 10.93 (10.53 - 11.33) |
| Northeast | 2016 | 10.53 (10.14 - 10.92) |
| Northeast | 2017 | 10.12 (9.74 - 10.50) |
| Northeast | 2018 | 10.46 (10.08 - 10.85) |
| Northeast | 2019 | 10.44 (10.06 - 10.82) |
| Northeast | 2020 | 9.94 (9.57 - 10.31) |
| Northeast | **2021** | 10.67 (10.28 - 11.07) |
| Northeast | **2022** | 10.29 (9.91 - 10.66) |
| Northeast | **2023** | 10.43 (10.04 - 10.81) |
| Northeast | **Total** | 10.12 (9.72 - 10.52) |
| Midwest | 1999 | 8.87 (8.49 - 9.26) |
| Midwest | 2000 | 8.65 (8.28 - 9.03) |
| Midwest | 2001 | 8.37 (8.01 - 8.74) |
| Midwest | 2002 | 8.64 (8.26 - 9.01) |
| Midwest | 2003 | 8.68 (8.31 - 9.04) |
| Midwest | 2004 | 8.62 (8.25 - 8.98) |
| Midwest | 2005 | 9.50 (9.12 - 9.89) |
| Midwest | 2006 | 8.93 (8.56 - 9.30) |
| Midwest | 2007 | 9.03 (8.66 - 9.39) |
| Midwest | 2008 | 9.36 (8.99 - 9.73) |
| Midwest | 2009 | 9.25 (8.89 - 9.62) |
| Midwest | 2010 | 9.27 (8.91 - 9.63) |
| Midwest | 2011 | 9.66 (9.30 - 10.03) |
| Midwest | 2012 | 9.77 (9.40 - 10.13) |
| Midwest | 2013 | 10.36 (9.99 - 10.74) |
| Midwest | 2014 | 10.27 (9.90 - 10.63) |
| Midwest | 2015 | 10.47 (10.10 - 10.84) |
| Midwest | 2016 | 10.44 (10.07 - 10.80) |
| Midwest | 2017 | 10.50 (10.14 - 10.87) |
| Midwest | 2018 | 10.66 (10.29 - 11.02) |
| Midwest | 2019 | 10.46 (10.10 - 10.82) |
| Midwest | 2020 | 10.54 (10.18 - 10.90) |
| Midwest | **2021** | 11.75 (11.35 - 12.14) |
| Midwest | 2022 | 11.08 (10.71 - 11.45) |
| Midwest | 2023 | 11.08 (10.70 - 11.45) |
| Midwest | **Total** | 9.77 (9.40 - 10.14) |
| South | 1999 | 7.09 (6.80 - 7.38) |
| South | 2000 | 7.13 (6.84 - 7.42) |
| South | 2001 | 6.99 (6.70 - 7.27) |
| South | 2002 | 7.20 (6.91 - 7.48) |
| South | 2003 | 7.23 (6.94 - 7.52) |
| South | 2004 | 7.20 (6.92 - 7.49) |
| South | 2005 | 7.29 (7.01 - 7.57) |
| South | 2006 | 7.27 (6.99 - 7.54) |
| South | 2007 | 7.11 (6.84 - 7.38) |
| South | 2008 | 7.00 (6.73 - 7.26) |
| South | 2009 | 7.03 (6.76 - 7.29) |
| South | 2010 | 7.31 (7.05 - 7.58) |
| South | 2011 | 7.24 (6.98 - 7.49) |
| South | 2012 | 7.27 (7.01 - 7.53) |
| South | 2013 | 7.22 (6.97 - 7.47) |
| South | 2014 | 7.04 (6.80 - 7.29) |
| South | 2015 | 6.97 (6.73 - 7.21) |
| South | 2016 | 6.83 (6.60 - 7.07) |
| South | 2017 | 7.20 (6.97 - 7.44) |
| South | 2018 | 6.90 (6.67 - 7.12) |
| South | 2019 | 7.06 (6.83 - 7.29) |
| South | 2020 | 6.87 (6.64 - 7.09) |
| South | **2021** | 7.58 (7.34 - 7.82) |
| South | **2022** | 7.45 (7.22 - 7.68) |
| South | **2023** | 7.43 (7.20 - 7.66) |
| South | **Total** | 7.16 (6.90 - 7.41) |
| West | 1999 | 10.28 (9.81 - 10.75) |
| West | 2000 | 10.81 (10.34 - 11.29) |
| West | 2001 | 10.29 (9.84 - 10.75) |
| West | 2002 | 10.41 (9.96 - 10.87) |
| West | 2003 | 10.58 (10.13 - 11.03) |
| West | 2004 | 10.60 (10.16 - 11.05) |
| West | 2005 | 11.27 (10.82 - 11.72) |
| West | 2006 | 11.09 (10.65 - 11.53) |
| West | 2007 | 11.35 (10.91 - 11.79) |
| West | 2008 | 10.98 (10.55 - 11.41) |
| West | 2009 | 11.47 (11.04 - 11.90) |
| West | 2010 | 11.67 (11.24 - 12.10) |
| West | 2011 | 11.24 (10.82 - 11.65) |
| West | 2012 | 11.13 (10.72 - 11.53) |
| West | 2013 | 11.75 (11.34 - 12.16) |
| West | 2014 | 10.91 (10.52 - 11.30) |
| West | 2015 | 11.01 (10.63 - 11.40) |
| West | 2016 | 11.17 (10.78 - 11.55) |
| West | 2017 | 10.64 (10.27 - 11.01) |
| West | 2018 | 10.62 (10.26 - 10.99) |
| West | 2019 | 10.67 (10.31 - 11.03) |
| West | 2020 | 10.15 (9.80 - 10.50) |
| West | **2021** | 11.34 (10.96 - 11.72) |
| West | **2022** | 10.56 (10.21 - 10.91) |
| West | **2023** | 10.39 (10.04 - 10.75) |
| West | **Total** | 10.90 (10.48 - 11.31) |

**Supplemental Table 7:** Aortic Stenosis and Heart Failure–related Mortality, Stratified by Place of Death in Older Adults in the United States, 1999 to 2023

| Place of Death | Number of Deaths |
| --- | --- |
| Medical Facility - Inpatient | 104,230 |
| Medical Facility - Outpatient or ER | 8,812 |
| Medical Facility - Dead on Arrival | 586 |
| Decedent's home | 74,254 |
| Hospice facility | 14,130 |
| Nursing home/long term care | 66,668 |
| Other | 12,241 |

**Supplementary Table 8: Comparison of Age - Adjusted Rate (95% CI) between Heart - failure and Aortic stenosis from 1999 - 2023**

|  | Age-Adjusted Rate (95% CI) | |
| --- | --- | --- |
| Year | **Heart Failure** | **Aortic Stenosis** |
| 1999 | 299.44 | 21.09 |
| 2000 | 299.24 | 21.2 |
| 2001 | 292.07 | 20.84 |
| 2002 | 286.65 | 20.71 |
| 2003 | 284.16 | 20.67 |
| 2004 | 275.36 | 20.59 |
| 2005 | 276.98 | 20.89 |
| 2006 | 261.96 | 20.55 |
| 2007 | 251.05 | 20.84 |
| 2008 | 249.5 | 21.36 |
| 2009 | 238.28 | 21.23 |
| 2010 | 238.08 | 21.83 |
| 2011 | 235.17 | 21.92 |
| 2012 | 230.7 | 21.77 |
| 2013 | 236.2 | 22.07 |
| 2014 | 237.68 | 21.75 |
| 2015 | 248.83 | 21.56 |
| 2016 | 248.25 | 21.3 |
| 2017 | 253.85 | 21.17 |
| 2018 | 258.13 | 20.35 |
| 2019 | 260.8 | 20.43 |
| 2020 | 281.95 | 20.49 |
| 2021 | 299.21 | 22.18 |
| 2022 | 284.45 | 21.14 |
| 2023 | 280.73 | 20.81 |
| Total | **260** | **21** |
